# Annotations

**Published:** 1887-04-02

**Authors:** 


### ANNOTATIONS.

**Endowed Beds.** THE people of Birmingham are famous for their business aptitude, and they justify the good opinion of their neighbours by enforcing sound business principles in the administration of their charities. The Jaffray Hospital is a case in point. Established quite recently, it now has an endowment fund rapidly approaching £40,000. To supplement this, the endowment of beds by individuals has been encouraged, with the result that five have already been so endowed with £1,250 each, producing interest equal to the annual cost of the bed. It is an open secret that the fifth bed was founded by Mr. John Jaffray, J.P., in memory of his daughter-in-law, whose death last year caused general regret. She was the daughter of Sir Francis Scott, of Great Barr Hall, and was widely popular in Birmingham and the neighbourhood. We have often advocated endowed beds, and we know no more affectionate, lasting, and wholly admirable mode of perpetuating the memory of a departed friend than such an endowment. There are various methods of endowment. Some are content to name a bed by giving a definite sum as a donation to the hospital. Others give annual larger amounts, and so acquire the right of naming an entire ward. But in our opinion the least ostentatious, and therefore the most satisfactory and practical system, is to pay in a capital sum to the bankers of the hospital selected, which, when invested at four per cent., will yield at least £50 per annum. The Birmingham people have favoured the last method, and we can only hope it may become widely popular. In any case, the managers of the various institutions might usefully call attention in their annual reports to the system of ward and bed endowments, and so get the idea presented to the supporters of hospitals in all parts of the country.

**Aberdeen and its Hospitals.** The inhabitants of Aberdeen will have to have a care, or their reputation will be permanently damaged. Not once, but often during the last twelve months, has the condition of its hospitals and their mismanagement caused surprise and astonishment in the minds of their countrymen, who have a knowledge of the facts. The Aberdeen City Hospital has long had an unenviable reputation for mismanagement, and the recent death of one of the nurses of the Children's Hospital, whilst under treatment there, has at last attracted so much attention to the abuses in question, that we cannot believe they will be allowed to continue longer unredressed. The special commissioner of the *Aberdeen Evening Gazette*, who has inspected the City Hospital, declares that "dirt and disorder were in the ascendant everywhere throughout the establishment. The reception block with its concrete floors is a disgrace to the City of Aberdeen, the wards are low, damp, and dirty, the ventilation is defective, a broken window was stuffed with paper and all were dirty, the lavatories sorely needed cleansing, the baths looked particularly filthy, and a considerable amount of cooking is done in each of the wards." Had such a description been written by a stranger it would have been set down to malice. It is, however, the report of a special local commissioner, who writes in no spirit of exaggeration, but with a serious sense of responsibility. In these circumstances we await the result of the inquest upon the unfortunate nurse we have referred to, and we cannot but believe that the people of Aberdeen will insist upon an immediate and searching inquiry, to be followed by the permanent abolition of the present disgraceful mismanagement and disorder.

**Chronic Hospitals.** It is interesting to note, in connection with the General Hospital, Birmingham, what the medical committee report with reference to the working and advantages of the first chronic hospital ever established in this country. The Jaffray Suburban Branch Hospital has proved a great boon, not only to the patients and their friends, but to the medical profession in Birmingham. One-third of the whole number of patients admitted during the year were discharged cured, and nearly one-half were relieved, many of them in a very material degree. It is noticeable that the proportion of deaths was considerable, and the medical committee report this fact to be due mainly to a desire on their part to give a last chance of improvement in desperate cases. In some instances that last chance had proved efficient in saving a patient's life, or in avoiding the necessity of serious surgical operations. In cases of chronic wounds, severe burns, and in joint diseases, most conspicuous benefit had resulted to the patients by their transfer from the General Hospital in the centre of the town to the Jaffray Suburban Branch Hospital. Another class of cases in which the chronic hospital has proved of great advantage is that of patients suffering from acute diseases, who, while insufficiently restored to be transferred to a convalescent home where medical attendance is not available, have been admitted into the Suburban Hospital and so greatly benefited. The fresh air undoubtedly hastened the recovery of the patients and rendered them more stable and healthy afterwards. The prolonged residence of chronic cases at the Jaffray Hospital has relieved the parent institution of much of the pressure from which it formerly suffered, and so many more patients who were acutely ill have been relieved within its wards. Mr. John Jaffary showed much commendable liberality by his generous gift to Birmingham, but the practical utility of that gift entitles him to even greater gratitude than the gift itself. It would be well if the committees of all large town hospitals were to make themselves acquainted with the objects and work of the Jaffray Suburban Hospital, Birmingham.

